# Supplementary material for: Mechanical analysis of eight-legged temporary support structure under wave forces
Source: PLoS One. 2023 Nov 29;18(11):e0277353. doi: 10.1371/journal.pone.0277353 (PMC10686482; doi:10.1371/journal.pone.0277353)
Supplement: Attachment — Submitted filename: Response to Reviewers1.docx [file pone.0277353.s001.docx]

Dear Editor and Reviewers

Thanks very much for taking your time to review this manuscript. I really appreciate all your comments and suggestions! Please find my itemized responses in below and my revisions in the re-submitted files. We have studied comments carefully and have made correction which we hope to meet with approval. Revised portions are marked in red in the paper. The main corrections in the paper and the responds to the reviewer’s comments are as flowing:

Reviewer 1：

Q:I don't understand why you study temporary structures rather than the permanent offshore structures? what's the difference between them?

A：Compared with the permanent pile foundation, the offshore wind turbine pile foundation drilling platform studied in this paper has a shallower rock-socketed depth, but the working stability requirement is higher than that of the permanent component pile foundation. Therefore, it is necessary to analyze the stability of this kind of structure.

Q:The variable should be italic in the paper, please modifying.

A: This article has revised the requirements of reviewers.

Q:In your study, the water depth is 20m with the wave height 5m, which seems impossible in reality. Please giving your explanation.

A: Thanks to the reminder of the reviewer, the working condition of this paper is extracted from the information of the working condition of Nanhai wind turbine pile foundation construction of Long Yuan Group. The lowest offshore altitude is 20 m, and the highest wave height is 5 m in strong typhoon weather, which is the worst working condition. This paper is taken as the calculation condition.

Reviewer 2：

Q:(1) first, I believe the English needs to be improved. The expression such as “control the fluid” is very rarely used in this field.

A: Thanks to the reviewer for reminding me that this article proof reads these words one by one.
Q:(2) the software used in this simulation has to be stated clearly.

A: This paper introduces the software introduced by the assessors.

Q:(3) the wave elevation time history shown in Fig. 3 is not satisfactory. The state-of-the-art CFD software can achieve very accurate wave field. Please check with your numerical model and see if your result can be improved.

A: Thanks to the reminder of the reviewers, the simulation software of this paper adopts FLAC-3 D finite difference method to model, and its software feature is that it can measure the mechanical change law of components under the condition of large deformation. This section is to verify the measurement of wave force elevation of shallow-buried structures by waves, because the experimental data used in this article are collected from the highest and lowest points of waves. Therefore, this paper makes some adjustments to the simulation model, and modifies the relevant calculation content.

Q:(4) the numerical model is only validated by the wave propagation problem as shown in Fig. 3, but I believe this is not enough, at least one example with structure included has to be shown and presented.

A: Thanks to the suggestion of the reviewers, Figure 3 was modified in this paper, and the schematic diagram of Mo-water tank test (Figure 3 a) was added to express related concepts.
